# Operational inefficiency and perceived quality in a paediatric day hospital: a lean-informed study of patient flow, caregiver and staff perceptions

**DOI:** 10.1093/intqhc/mzag087

**Published:** 2026-06-19

**Authors:** Mario Casolino, Lavinia Benedetti, Giovanni Gioiello, Daniele Mascia, Martina Vardabasso

**Affiliations:** Institute for Maternal and Child Health—IRCCS “Burlo Garofolo”, Trieste, 34137, Italy; Institute for Maternal and Child Health—IRCCS “Burlo Garofolo”, Trieste, 34137, Italy; Department of Medicine and Surgery, University of Enna “Kore”, Piazza dell’Università, Enna, EN 94100, Italy; Luiss Libera Università Internazionale degli Studi Sociali Guido Carli, Roma, 00198, Italy; Institute for Maternal and Child Health—IRCCS “Burlo Garofolo”, Trieste, 34137, Italy

## Abstract

**Background:**

Lean management is widely promoted in healthcare to improve efficiency; however, evidence is sparse concerning how Lean-informed organisational settings directly influence patient experience and frontline staff perceptions of Lean processes, particularly in paediatric day hospital (DH) environments where clinical complexity and family-centred care create inherent conflicts between standardisation and adaptability.

**Methods:**

We conducted an observational cross-sectional study using several quantitative data sources, supplemented by qualitative insights from semi-structured staff interviews in an Italian paediatric DH following staff introduction to Lean concepts (Sept–Nov 2024). We integrated: (i) Value Stream Mapping and timed caregiver tracking to evaluate patient flow and waiting; (ii) a caregiver satisfaction survey at discharge; and (iii) a staff survey plus interviews capturing perceptions of Lean. Analyses were descriptive and exploratory.

**Results:**

*Process performance:* The DH exhibited substantial variation; only 11.4% of procedures occurred precisely on schedule, and non–value-added time constituted approximately 65% of each stay. *Patient experience*: Despite these inefficiencies, caregiver satisfaction remained consistently high (median 5/5). Satisfaction was only weakly correlated with waiting times or schedule deviations but strongly associated with transparent information and respectful staff interactions. *Staff perceptions:* The majority viewed Lean as practical and valuable, especially among those previously trained; however, many cited obstacles such as insufficient managerial support and resistance to change, with older staff expressing more scepticism than younger colleagues.

**Conclusions:**

High caregiver satisfaction coexisted with considerable operational inefficiencies, suggesting that relational care mitigates the impact of delays. These findings demonstrate that efficiency metrics alone do not fully represent perceived quality in paediatric settings. Lean implementation should balance efficiency, clinical adaptability, and family-centred care.

## Introduction

Lean management is a philosophy focused on enhancing value by eliminating waste and streamlining processes. Developed through the Toyota Production System, Lean has been increasingly applied in healthcare settings [1]. Since the early 2000s, adoption has accelerated as healthcare faces ageing populations, rising costs, limited resources, workforce shortages, and challenges intensified by the COVID-19 pandemic [2, 3]. Lean methods are used to improve productivity and quality while controlling costs, enabling systems to operate effectively despite tight constraints [4]. The main takeaway is that Lean management helps healthcare systems manage limited resources while maintaining quality and productivity.

In healthcare, Lean initiatives aim to identify and eliminate waste, defined as activities that do not add value from the patient’s perspective, with the objective of reducing delays, improving process flow, and boosting staff motivation [5–8]. Studies have linked Lean adoption to increases in patient satisfaction, shorter waiting times, better safety, and improved service quality [5, 9–11]. However, recent critiques note that many Lean applications in hospitals prioritise internal efficiency and technical process optimisation, sometimes at the cost of system-level responsiveness, external effectiveness, and the experiential aspects of care [12, 13].

Evidence shows that successful Lean adoption relies not only on technical tools but also on organisational culture, a continuous-improvement mindset, and active staff engagement [2]. Limited staff participation is widely recognised as a significant barrier to Lean implementation, given the central role of human, relational, and behavioural factors in healthcare delivery [14, 15]. Recent studies also emphasise the value of sense-making processes and psychological safety among healthcare professionals during complex organisational change, yet operational aspects such as scheduling and frontline engagement are rarely examined [16].

So far, Lean research in healthcare has mainly examined high-throughput or high-acuity settings, including emergency departments [17, 18], operating rooms [19], and radiotherapy centres [8].

Conversely, empirical evidence on Lean in paediatric day hospital (DH) settings remains sparse and fragmented, as most studies address paediatric care more broadly instead of Lean implementation in single-day, multidisciplinary pathways [20, 21, 22]. This evidence underscores the lack of specific research on Lean in paediatric DH.

The paediatric DH is a unique and complex organisation for three reasons: caring for children with rare or chronic conditions is medically challenging; there is a need for tight coordination across multiple specialised services; and publicly funded systems impose structural constraints, such as limited staffing and financial resources [23], which may be further exacerbated by regional workforce distribution disparities within national health systems [24].

Delivering family-centred care is essential in paediatrics, as caregiver experience directly affects treatment adherence and care outcomes. Paediatric patients also have unique developmental, emotional, and psychosocial needs that require specialised care pathways, distinct from those used for adults [25].

From a methodological perspective, Lean studies frequently rely on process optimisation tools such as Value Stream Mapping (VSM), Spaghetti Plots, and Rapid Improvement Events [26, 27]. Despite these methods, patient-reported satisfaction and experience measures are not consistently integrated into these analyses [28].

Where patient experience is considered, it is often secondary to operational indicators. Consequently, this leaves a limited understanding of how process inefficiencies interact with caregivers’ perceptions of quality.

Patient satisfaction is an important part of healthcare quality. In paediatrics, family views help build trust, support treatment, and sustain care [29, 30].

In paediatric DHs, where teams consolidate multiple consultations and procedures into a single day, service quality depends not only on clinical outcomes but also on relational, environmental, and organisational factors [31, 32, 22] that interact across each step of the care pathway [33, 22].

Recent studies suggest that families may report high satisfaction even in the presence of scheduling delays or fragmented workflows, largely due to positive interpersonal interactions and perceived staff dedication [34].

Quality can be seen in three levels of growth: following standards, meeting expectations, and going beyond by offering personal care [35]. This view aligns with patient-centred models, where feelings and experience are key to quality.

Bezerra’s framework identifies four core dimensions of healthcare service quality—physical environment, interaction, service outcome, and waiting time—which are particularly salient in paediatric DH settings, where families navigate complex, same-day, multidisciplinary pathways. The interaction among these dimensions—such as how empathetic communication can offset long waiting times—illustrates the inherent tension between efficiency and experience in dynamic clinical environments [32].

Despite increased interest in Lean management and healthcare service quality, empirical integration of these fields is rare. Specifically, limited evidence exists on how Lean-driven organisational contexts impact: (i) caregiver satisfaction, (ii) frontline staff perceptions, and (iii) patient flow performance concurrently, particularly in paediatric DH settings.

Within this framework, this study documents and analyses patient flow within the Paediatric DH of the IRCCS Burlo Garofolo using VSM to describe care pathways and pinpoint organisational bottlenecks [36]. The investigation also assesses caregiver experience and healthcare professionals’ perspectives to define workflow performance and support for quality care in a Lean-driven organisational context, without evaluating a formal Lean intervention.

### Research questions

The analysis explores interactions between operational performance, caregiver experience, and healthcare professionals’ perceptions in a Lean-informed paediatric DH.

Accordingly, the study addresses the following research questions:

**RQ1:** *How do delays and schedule deviations in a paediatric Day Hospital affect caregivers’ satisfaction?*

**RQ2:** *How do healthcare professionals view Lean management after learning Lean concepts and participating in related activities in the Paediatric Day Hospital? What barriers and facilitators influence these views?*

This study offers two complementary contributions to the Lean healthcare literature. First, it provides empirical evidence on how operational performance, caregiver experience, and staff perceptions—defined respectively as care process efficiency, caregiver satisfaction, and staff attitudes—interact within a Lean-informed paediatric DH, an integration rarely examined in existing studies. Second, it addresses a clear gap by focusing on paediatric DH pathways, combining process metrics, patient-reported experience, and staff perspectives in a single cross-sectional study drawing on multiple quantitative data sources and qualitative insights from interviews. Rather than testing the effectiveness of Lean, the study generates evidence to inform the design of future improvement initiatives and evaluative research.

## Materials and methods

### Setting

The study was undertaken at the Institute for Maternal and Child Health—IRCCS “Burlo Garofolo” (Trieste, Italy), a tertiary paediatric and maternity centre within the Italian National Health Service. Like the English NHS, it is predominantly publicly funded. The Paediatric DH manages approximately 1,000 inpatient admissions and 22,000 outpatient visits annually. Its multidisciplinary services include endocrinology, rheumatology, allergy, nephrology, and rare metabolic diseases. Care delivery integrates outpatient consultations with same-day hospitalisations. Procedures include blood collection, intravenous therapies, and semi-invasive interventions. Despite a decade-long reduction in staffing, service volumes have increased, placing greater operational strain and highlighting the need for advanced management strategies.

The DH is a busy and complex place where patient flow, scheduling, and teamwork are key to good performance.

### Study design, organisational context and exposure to lean concepts

This observational diagnostic study, reported in accordance with the SQUIRE 2.0 guidelines [37], was designed as a cross-sectional assessment conducted between September and November 2024.

The study combined multiple quantitative data sources with qualitative insights from semi-structured staff interviews. This approach provided a comprehensive understanding of organisational processes, caregiver experience, and staff perceptions within a Lean-informed context.

Rather than evaluating the effects of a formal intervention, the study documents organisational processes and staff perceptions following exposure to Lean concepts.

Two sequential managerial activities framed the context of the study:

1. Exposure to Lean concepts through targeted training for a multidisciplinary “Lean Team”.
2. A locally driven review of selected patient pathways within the Paediatric DH.

The Lean initiative at the DH began in September 2024. A multidisciplinary “Lean Team” (physicians, nurses, and allied professionals) received structured training from external experts on core Lean principles, such as process mapping (visualising workflows), waste identification (recognising inefficiencies), and collaborative problem-solving (working together to find solutions). Training combined theoretical sessions with case-based exercises. Content was tailored to local needs identified through preliminary staff interviews. There was particular attention to change management, managing resistance, and building a shared vision for continuous improvement.

After training, the Lean Team helped map patient pathways using flowcharts, from appointment scheduling to same-day discharge. Shared digital tools, including a cloud repository and structured e-mail updates, improved transparency and collective learning. These activities were not formal interventions but set the organisational context for this diagnostic study. Data collection for this analysis took place from September to November 2024, during routine service delivery.

### Data sources and instruments

This study drew upon multiple sources of evidence to address its aims. Quantitative data included time-tracking measures of patient flow, caregiver-reported satisfaction, and staff survey responses. These were complemented by qualitative insights from staff interviews, which provided contextual understanding of organisational processes and perceived barriers to Lean implementation.

### Staff interviews

Semi-structured interviews (*n* = 16) were conducted with a purposive sample including the DH nurse coordinator, two nurses responsible for appointment scheduling and activity planning, three bedside nurses providing direct patient care, one nurse manager, and physicians representing each DH specialty. Participants were selected based on role, seniority, and experience to ensure representation of all key clinical and organisational areas of the DH.

Building on participant selection, the interviews aimed to: (i) map patient pathways from admission to discharge; (ii) identify inefficiencies and bottlenecks; (iii) explore sources of operational waste; and (iv) elicit staff perspectives on barriers and enablers to Lean adoption. Throughout, recurring issues included communication breakdowns, delays in patient flow, and redundant or non-value-adding processes.

These qualitative interview data subsequently informed process mapping and contextual interpretation, rather than undergoing formal qualitative analysis (for example, coding or thematic analysis).

### Patient questionnaire and time-tracking instrument

**Figure 1:**
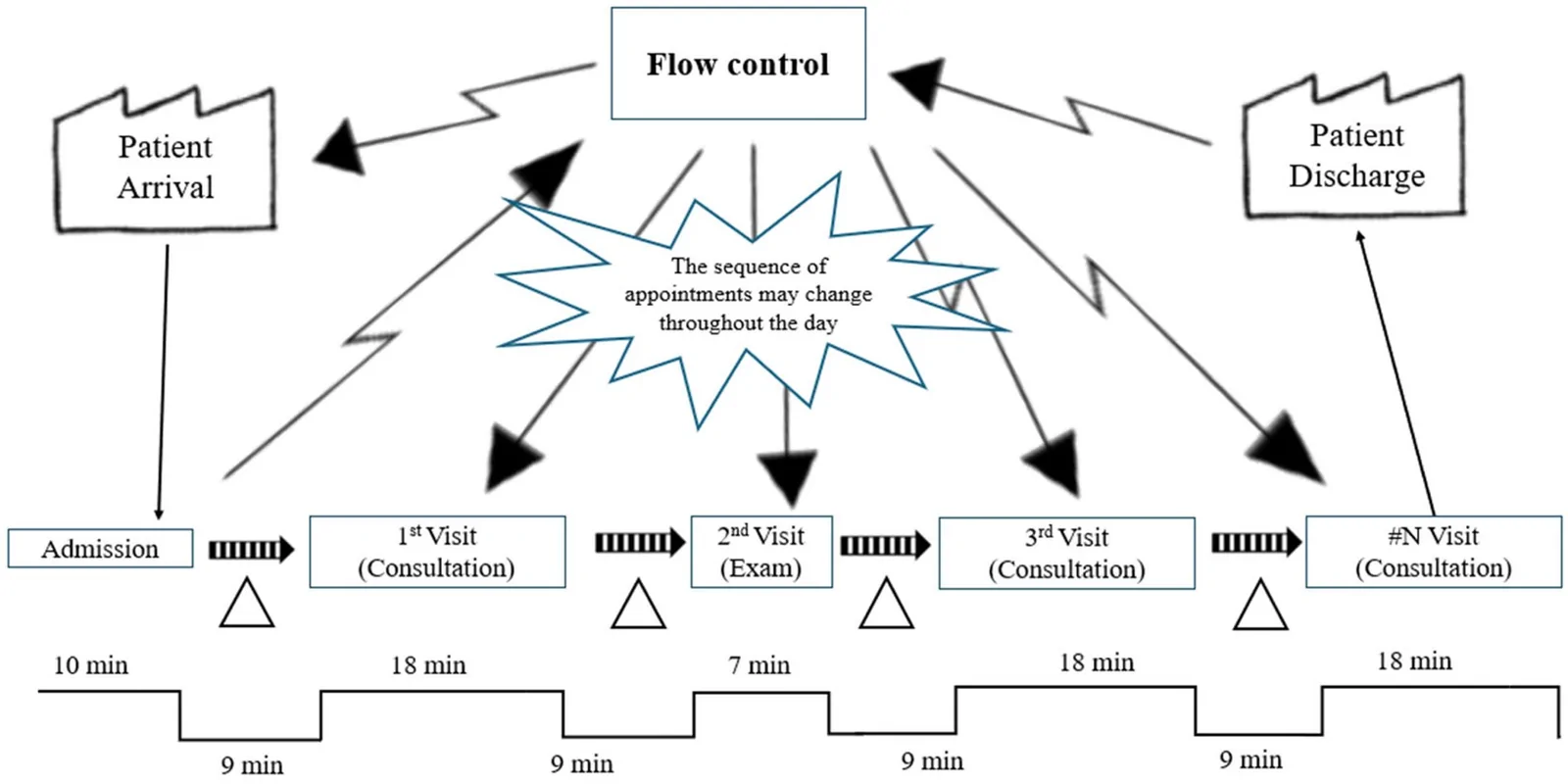
Patient pathway in the department—VSM. On admission day, after check-in, the nursing staff sets the sequence of consultations and examinations. Patients complete evaluations and are discharged the same day. Time indications refer to median visit, examination, and waiting durations. VSM, Value Stream Mapping.

A structured patient–caregiver questionnaire was administered and comprised of two integrated components:

1. A time-tracking instrument (process data).
  Because the hospital system could not capture detailed waiting times or deviations from scheduled appointments, a custom time-tracking tool was included in the questionnaire. Caregivers received brief instructions from reception nurses at admission on how to record key time points along their child’s pathway, such as:
  - scheduled time of consultation/exam;
  - time at which staff notified caregivers to proceed to the consultation room;
  - time of arrival in front of the room;
  - start and end time of consultation/exam;
  - time of return to the DH waiting area.
  Caregivers reported some timestamps, while scheduled times and admission times were recorded by reception staff to enhance accuracy. This distinction highlights the transition from self-reported to staff-recorded data.
  Using these timestamps, the team calculated length of stay (LOS), waiting times, consultation durations, and deviations from schedule. This transition allowed distinction between value-added (VA) and non-value-added (NVA) activities based on Lean definitions [5].
  VA time was defined strictly as the actual duration of medical consultation or execution of a clinical procedure/examination.
  NVA time included all waiting intervals, administrative steps, transfers between areas, and idle periods before discharge to ensure clarity and usability of the instrument, the research team developed, validated, and pilot-tested it with approximately 30 caregivers in July–August 2024 across clinical pathways before full deployment in September 2024.
  Based on interviews and the first part of questionnaire data, patient journeys were mapped using VSM to trace the pathway from hospital check-in through medical consultations and diagnostic examinations to same-day discharge (Fig. 1).
  VSM and patient flow data were collected after staff exposure to Lean concepts, but before any formal process redesign. This defined the transition to documenting the organisational state after Lean exposure, rather than evaluating it after intervention.
2. Patient satisfaction scale
  The survey assessed satisfaction and perceptions of delay, addressing a gap in Lean studies that often overlook patient experience [28]. Because patient-perceived value is influenced by service efficiency and clinical outcomes [34], the questionnaire specifically examined how throughput time relates to satisfaction.
  The satisfaction section was built on Bezerra’s framework of healthcare service quality [31], assessing four dimensions: physical environment, interaction quality, service outcome, and waiting time. Items were scored on a 1–5 Likert scale (1 = not at all satisfied; 5 = very satisfied) and covered reception at admission, staff courtesy, clarity of information, privacy, signage, cleanliness, organisation of services and overall satisfaction.
  The patient satisfaction questionnaire was also content-validated by the research team, drawing on clinical and methodological expertise, to ensure item relevance and clarity. It was pilot-tested from July to August 2024 with approximately 30 caregivers across different clinical pathways to assess feasibility, comprehension, and completeness, resulting in minor refinements before full deployment.
  At DH admission, reception nurses provided caregivers with the questionnaire and clear instructions for the time-tracking section. Caregivers kept the instrument, recording real-time time points during the stay (Part 1), then completed the satisfaction section (Part 2) before discharge.

### Staff perception survey

A structured survey was administered using REDCap, a secure hospital platform, with three sections: (i) effectiveness of Lean (6 items); (ii) barriers to adopting Lean (7 items); and (iii) interest in Lean (3 items). Items were rated on a 7-point Likert scale (1 = strongly disagree; 7 = strongly agree) [38].

The instrument was developed by our research team and internally validated for content and face validity through a pilot study with 15 DH professionals, ensuring clarity and relevance prior to full administration in September 2024.

The survey was distributed to all professionals regularly working in the DH, including physicians, nurses, healthcare assistants, and resident physicians.

### Statistical methods

Quantitative variables were summarised using medians and interquartile ranges (IQR) for ordinal data, and frequencies (%) for categorical variables. Associations between waiting times, schedule deviation, and patient satisfaction were examined with ordinal logistic regression models. Associations between staff characteristics (age, prior Lean training) and Lean perceptions were explored using linear regression models.

Model assumptions (linearity, proportional odds, homoscedasticity, and independence) were assessed before analysis. All analyses were two-sided with significance set at *P* < .05, and 95% confidence intervals (95% CI) were reported. Analyses used Stata 18.5 (StataCorp, College Station, TX, USA). With a cross-sectional design, regression analyses were interpreted as exploratory and associative. No causal inferences were drawn.

## Results

### Patient characteristics

A total of 513 patient questionnaires were distributed, with 443 returned (response rate 86.4%). The median age was 11 years (IQR 7–14), and 44.9% were male. Patient demographics, place of residence, type of visit, and medical speciality are reported in Table 1.

**Table 1:** Patient characteristics and percentage distribution of medical specialty.

	*n* = 443
Age, median (IQR)	11 (7–14)
Female gender (%)	45.1%
Place of residence, (%)	
Same city of the hospital	35.9%
<100 km from the hospital	50.8%
>100 km from the hospital	13.3%
Type of visit, (%)	
Outpatient	67.5%
Inpatient (day hospital)	32.5%
Reason for access, (%)	
First-time visit	6.1%
Follow-up visit	90.5%
Other	3.4%
Children by medical speciality (%)	
Endocrinology	50.1%
Rheumatology	18.6%
Rare diseases	17.7%
Nephrology	8.3%
Neuropsychiatry	2.9%
Immunology	1.6%
Allergology	0.9%

Data are presented as median (IQR) for age, and as number (percentage) for all other variables; IQR = interquartile range; (%): percentage relative to the total (*n* = 443).

The sample was representative of the DH case-mix based on 2023 activity, which totalled approximately 7,500 cases.

### Operational performance

**Figure 2:**
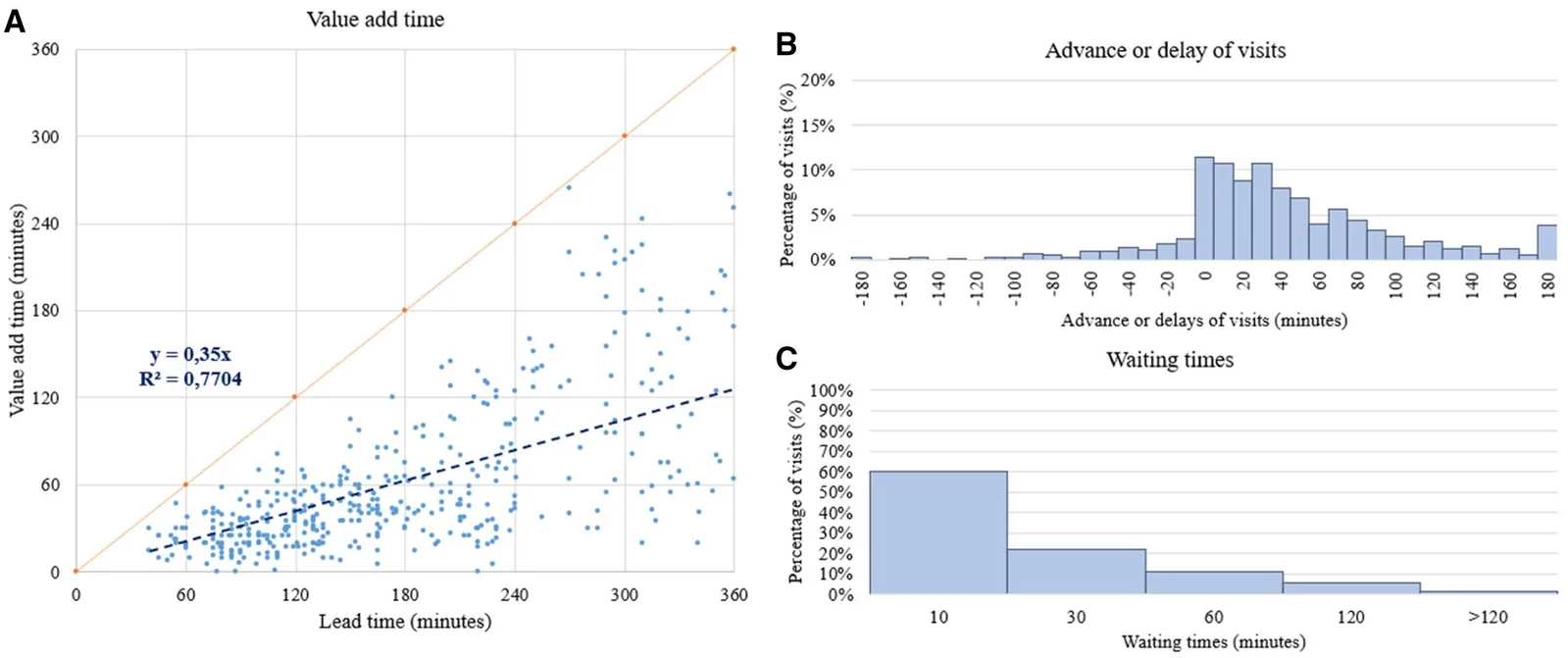
Operational performance. (A) Each patient’s lead time and value-added (VA) time are shown. The dotted line marks the regression model. The orange line shows the ideal scenario where lead time matches VA time. (B) Visit advances and delays are distributed. The x-axis shows minutes before or after schedule. The y-axis shows the percentage of visits in each interval. (C) Median waiting time at the clinic. The x-axis displays the time patients waited outside the clinic door after notification/call, ranging from 10 minutes to over 120 minutes. The y-axis shows the percentage of visits for each waiting time category.

The median lead time was 165 minutes (IQR 110–238). VA time, as time spent in consultation or procedures, was 35% of the total stay; 65% was NVA waiting time. Scheduling impacts efficiency, as seen in Fig. 2.

Admissions were concentrated between 08:30 and 10:00, while consultations lasted until about 13:00; no visits started at the scheduled 08:00 slot. The median deviation was 30 minutes (IQR 9–73); only 11.4% of procedures occurred exactly on schedule. These figures illustrate schedule-adherence patterns that affect operational efficiency.

The main insight from semi-structured interviews was the reconstruction of patient pathways and identification of key bottlenecks and inefficiencies, such as communication gaps and delays. These conclusions helped interpret time-tracking data and ensured the VSM reflected real workflows. Consequently, interview findings provided essential context for understanding observed schedule adherence.

Despite schedule variability, the median waiting time outside consultation rooms was 9 minutes (IQR 0–25), with 60% of patients waiting less than 10 minutes. This observation intertwines with broader patterns in patient stay: subjects residing more than 100 km from the hospital had longer stays (median 205 vs. 156 minutes), likely reflecting organisational strategies that cluster multiple procedures within a single visit for families travelling long distances.

### Patient satisfaction

**Figure 3:**
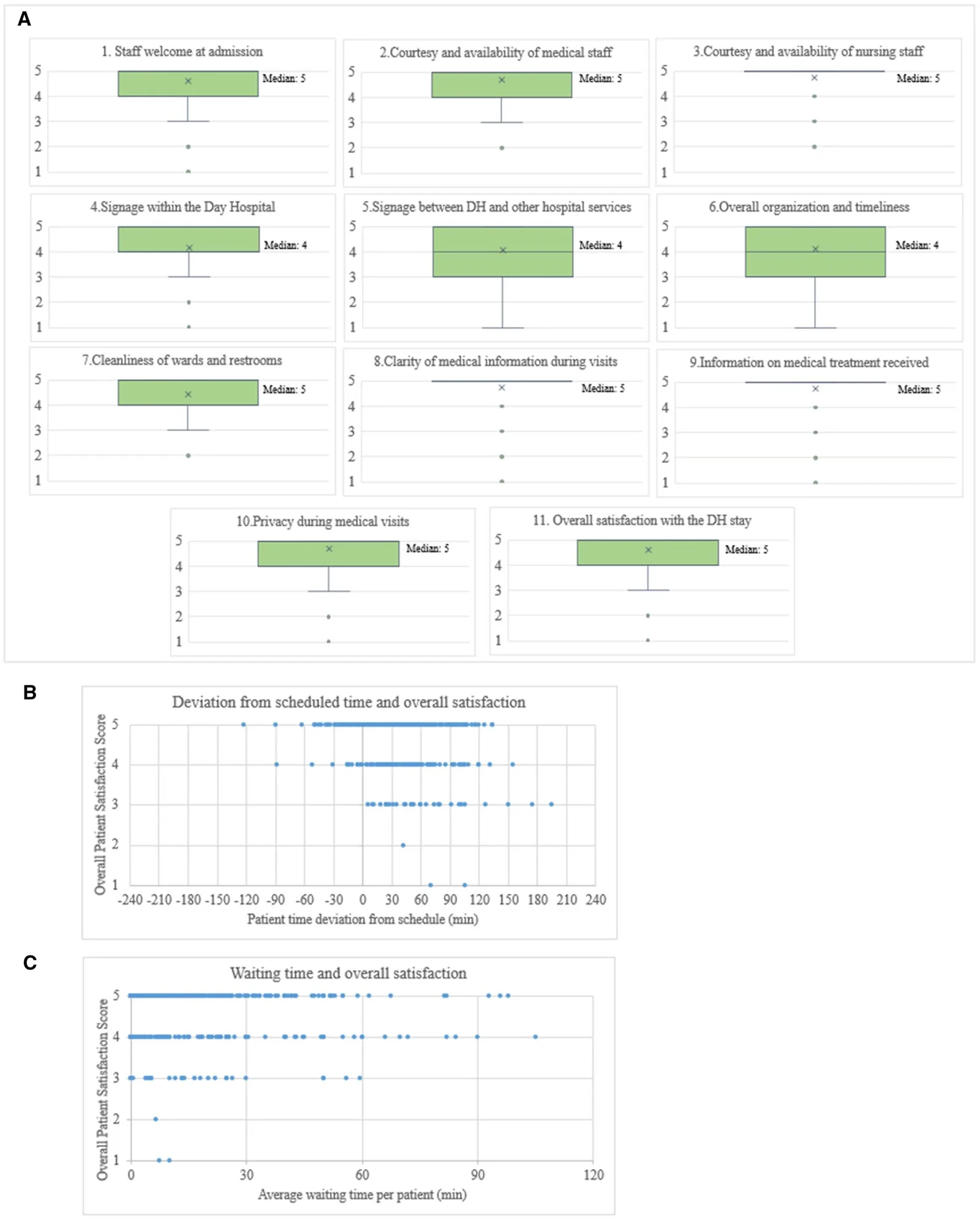
Patient satisfaction. (A) 11 box plots illustrating the distribution of family satisfaction levels across distinct dimensions; each box plot displays the median, interquartile range, mean, and identifies any outliers. The 11th box plot represents the overall satisfaction level. (B) The scatter plot shows the deviation from the scheduled visit time on the x-axis and overall patient satisfaction on the y-axis. It illustrates that overall patient satisfaction tends to decrease as the deviation from the scheduled time increases. (C) The scatter plot displays the average waiting time per patient on the x-axis and overall patient satisfaction on the y-axis. It shows a tendency for satisfaction to decrease as the average waiting time increases.

Overall patient satisfaction was high, with a median score of 5 out of 5 (Fig. 3).

The concentration of satisfaction scores at the upper end was evident, indicating a ceiling effect that may have reduced variability and limited regression sensitivity. Exploratory ordinal regression analyses (Fig. 3B) demonstrated that larger deviations from scheduled appointment time (OR = 0.996; *P* = .016) and longer waiting times (OR = 0.993; *P* = .030) corresponded with slightly lower odds of reporting the maximum satisfaction score. These associations should be interpreted cautiously given the limited outcome variability.

Higher satisfaction scores were observed among patients with clear medical information (OR = 7.64; *P* < .001), courteous interactions with physicians and nurses (OR = 12.19 and OR = 12.36; both *P* < .001), and positive admission reception (OR = 8.39; *P* < .001).

### Healthcare staff perception of Lean management

Among 144 healthcare professionals surveyed, 123 responded (response rate: 85.4%). A description of staff characteristics is presented in Table 2.

**Table 2:** Profile of healthcare personnel from the Department of Paediatrics.

	*n* = 123
Age, median (IQR)	46 (35–56)
Female gender, *n* (%)	84 (68.3%)
Professional qualification, *n* (%)	
Nurse	43 (35.0%)
Physician	28 (22.8%)
Resident	23 (18.7%)
Healthcare Assistant	11 (8.9%)
Healthcare Technician Staff	12 (9.8%)
Other	6 (4.9%)
Professional role, *n* (%)	
Clinical	102 (82.9%)
Managerial	9 (7.3%)
Administrative	7 (5.7%)
Other	5 (4.1%)
Years of work in the current department, *n* (%)	
<5 years	46 (37.4%)
5–10 years	27 (22.0%)
10–15 years	33 (26.8%)
15–20 years	10 (8.1%)
>20 years	7 (5.7%)
Years of work at IRCCS Burlo Garofolo, *n* (%)	
<5 years	36 (29.3%)
5–10 years	25 (20.3%)
10–15 years	29 (23.6%)
15–20 years	9 (7.3%)
>20 years	24 (19.5%)
Educational qualification, *n* (%)	
Middle or high school diploma	19 (15.4%)
Bachelor’s degree	20 (16.3%)
Master’s degree	49 (39.8%)
Specialisation/PhD	35 (28.5%)
Years since completion of basic degree, *n* (%)	
<5 years	32 (25.9%)
5–10 years	8 (6.5%)
10–15 years	20 (16.3%)
15–20 years	27 (21.9%)
>20 years	36 (29.3%)

Data are presented as median (IQR) for age, and as number (percentage) for all other variables; IQR = interquartile range; n: absolute number; (%): percentage relative to the total (*n* = 123).

**Figure 4:**
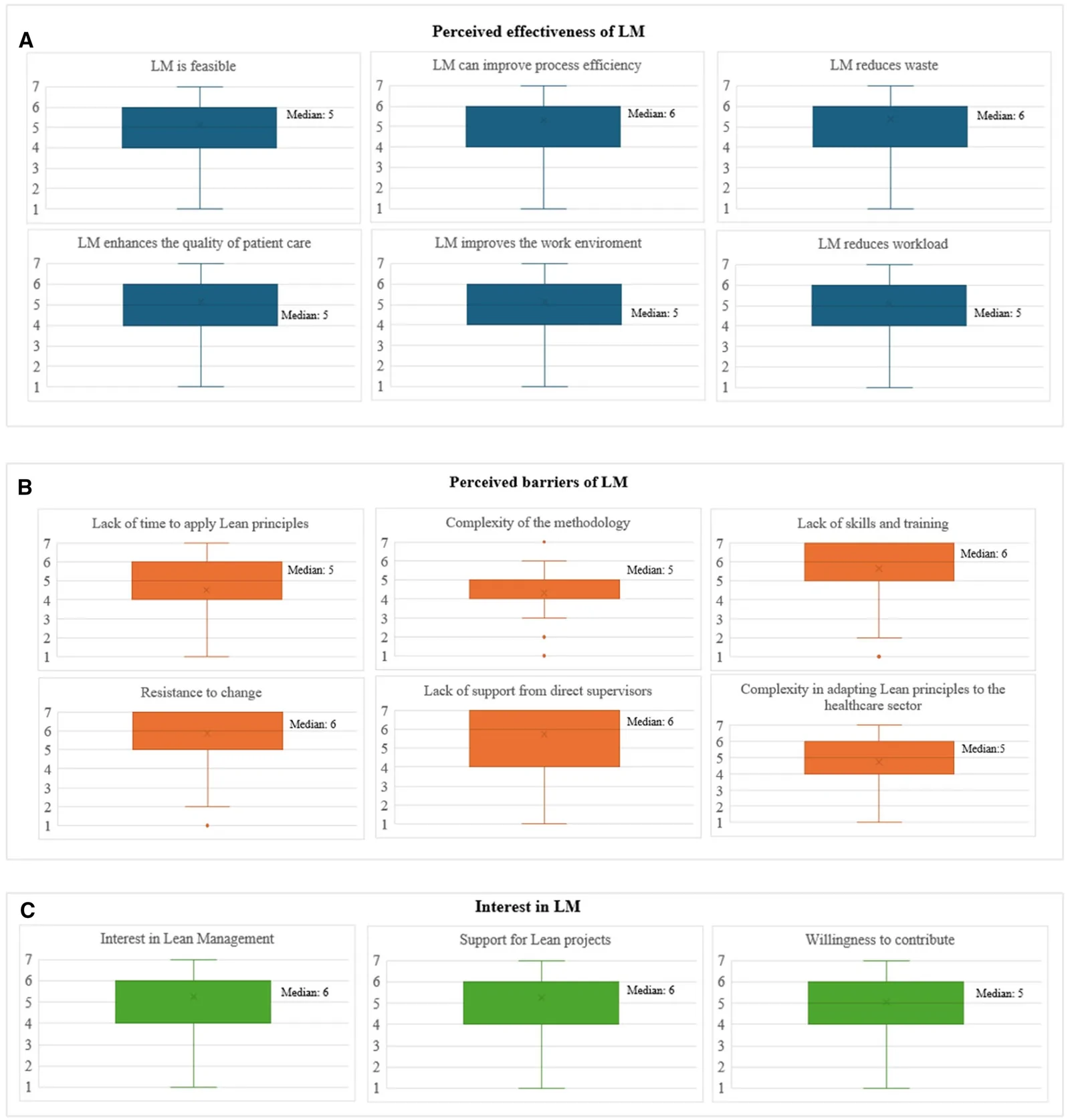
Healthcare staff perceptions of Lean management. The figure presents box plots depicting healthcare staff perceptions of Lean management effectiveness (A), identified obstacles (B), and interest in Lean projects (C). Each box plot displays the interquartile range, median, mean, and outliers.

After describing staff characteristics in Table 2, we found that approximately 60% of respondents reported familiarity with Lean, and 22.8% had received formal Lean training. Lean was generally perceived as feasible (median 5, IQR 4–6), potentially useful for improving efficiency (median 6, IQR 4–6), and relevant for reducing process waste (median 6, IQR 4–6). Reported barriers included resistance to change, limited training opportunities, perceived insufficient managerial support, and methodological complexity. Interest in Lean was high (median 6, IQR 4–6). Item-level responses are shown in Fig. 4.

Exploratory linear regression models indicated that staff with prior Lean training scored, on average, +0.9 to +1.1 points higher (on a 7-point scale) in perceived effectiveness than untrained staff (*P* = .005). Skilled staff also demonstrated +0.9 to +1.2 points higher interest in Lean (*P* < .001), and perceived barriers were 0.6 to 0.9 points lower among trained staff (*P* = .029).

Further examining demographic influences, professionals aged ≥45 years reported significantly lower interest and perceived effectiveness than younger colleagues (both *P* < .05) and were less likely to be familiar with Lean (55.6% vs. 82.4%; *P* < .001). Age was therefore significantly associated with Lean perceptions.

**Figure 5:**
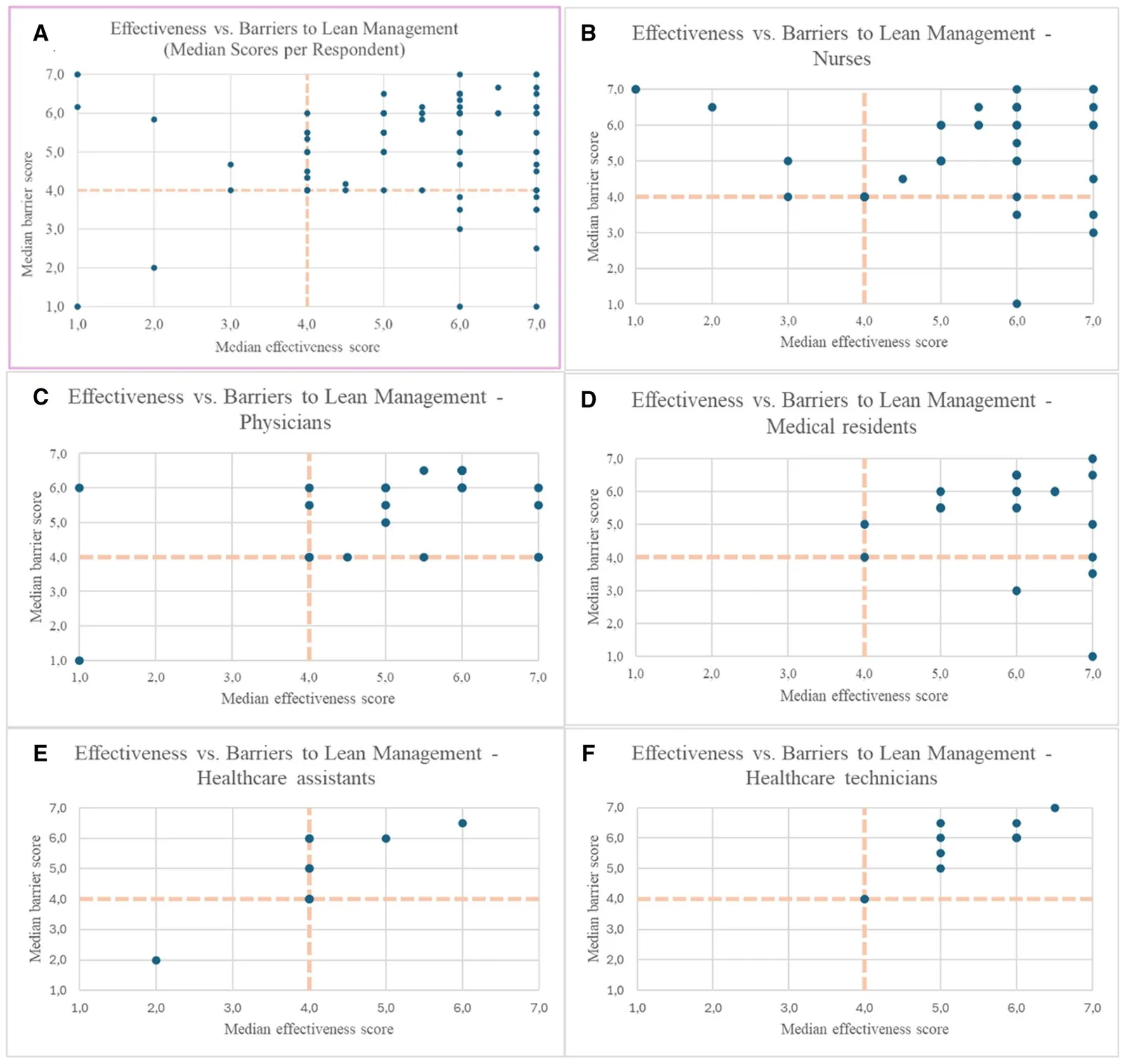
Scatter plot analysis. In graph (A), for each respondent, the median score for perceived barriers (on the y-axis) and the median score for Lean management effectiveness (on the x-axis) were calculated. Dashed lines indicate the neutral midpoint of the response scale. In the subsequent graphs, data are aggregated and displayed by professional role, including nurses (B), physicians (C), medical residents (D), healthcare assistants (E) and healthcare technicians (F).

Finally, the inverse relationship between perceived effectiveness and perceived barriers is illustrated in Fig. 5.

## Discussion

### Statement of principal findings

This study investigated associations between operational performance, caregiver satisfaction, and healthcare professionals’ perceptions in a paediatric DH implementing Lean-informed practices. Three key findings emerged.

First, the DH exhibited substantial operational inefficiencies: only 11.4% of procedures occurred at the scheduled time, the median schedule deviation was 30 minutes, and NVA time accounted for approximately 65% of the total length of stay. These figures indicate marked variability in patient flow and limited adherence to the schedule.

Second, despite these inefficiencies, caregiver satisfaction remained consistently high (median 5/5). Exploratory regression analyses revealed only modest negative associations between longer waiting times or greater schedule deviations and maximum satisfaction scores. In contrast, strong positive associations were observed with clear communication, courteous interactions, and quality of reception at admission, suggesting that relational and communicative aspects of care played a more decisive role in shaping caregiver experience than throughput efficiency alone. Third, staff perceptions of Lean were generally positive. Professionals with prior Lean training and younger staff reported higher perceived effectiveness and stronger interest in these procedures, whereas older staff tended to report more barriers.

Taken together, these outcomes address the two research questions: (i) operational inefficiencies were only weakly associated with caregiver satisfaction, which remained high due to strong interpersonal care; and (ii) staff perceptions of Lean were generally positive but stratified by age and prior training, with concerns centred on change management and managerial support.

### Interpreting the key paradox: high satisfaction despite inefficiency

Families reported high satisfaction despite considerable inefficiencies and schedule variability. Several complementary explanations merit discussion.

Clear communication and strong relationships overcame organisational issues. Satisfaction was mainly driven by clear medical information and courteous staff. In this DH, staff managed delays with attentive communication, reassurance, and personalised interactions.

Families at a tertiary paediatric centre for complex cases expect delays and complexities. Their satisfaction often reflects gratitude for specialised care rather than timely service. So, high satisfaction does not equal optimal organisational performance.

Third, some variability labelled as “NVA” time may represent necessary flexibility rather than avoidable waste. Paediatric DH care involves unpredictable clinical needs, emotional support for children and caregivers, and coordination across multiple specialties. These elements can legitimately extend time in care without constituting poor performance. Thus, not all deviations from schedule should be viewed as inefficiency requiring elimination; some may be intrinsic to family-centred care.

This interpretation balances efficiency with adaptability. Improvement efforts should balance standardisation and flexibility to meet clinical and psychosocial needs.

### Interpretation within the context of the wider literature

Our findings confirm that patient satisfaction depends mainly on communication quality, staff courtesy, and perceived respect, even in operationally constrained settings [39, 40]. Therefore, relational care can buffer the negative impact of delays on patient experience.

This study also extends Lean healthcare literature by focusing on a paediatric DH, a setting rarely examined in depth. While prior Lean studies have predominantly targeted emergency departments, operating theatres, and radiology units, our findings demonstrate that paediatric DHs present distinct organisational challenges that require tailored Lean approaches.

Additionally, unlike most previous analyses that examine process efficiency or patient experience in isolation, this investigation integrates operational performance, caregiver satisfaction, and staff perceptions into a single analytical framework. This combined perspective offers novel and more comprehensive insights into how efficiency and perceived quality interact in complex paediatric settings, highlighting the need to balance standardisation with flexibility in family-centred care.

Most examinations demonstrate a negative correlation between increasing waiting times and overall satisfaction (perceived quality, trust, and willingness to recommend the service) in outpatient, emergency department, and inpatient settings [41–43], although this relationship is not always consistent. The literature indicates that when communication quality from healthcare staff is high, differences in satisfaction between those reporting “long waits” and those who do not are minimal [44]. The subjective perception of waiting (how long it feels relative to expectations) appears to weigh more than the actual elapsed time [45, 46].

Regarding staff perceptions, younger and more highly trained staff reported greater perceived effectiveness and interest in Lean, whereas older staff expressed more reservations and perceived more barriers. However, these age-related differences should be interpreted with caution. Empirical evidence suggests that chronological age is not a primary determinant of Lean perceptions. For example, a large Canadian study found no significant associations between age group and attitudes towards Lean, including support and assumed value [47]. Similarly, psychometric tools developed to measure perceptions of Lean adoption have been validated on samples with wide age variability without showing systematic differences attributable to age [48].

Some evidence suggests a possible role of age, but with limited effects. Specifically, the study by Veres *et al.* [49] shows that age, along with other factors, may help explain the use of Lean practices and perceptions of waste. However, its contribution remains partial and secondary compared to other variables, such as knowledge of Lean and organisational role.

Overall, these findings suggest that, although viewed differences between age groups may emerge, attitudes towards Lean depend mainly on organisational and professional factors, such as role, workload, level of engagement, and quality of implementation. Furthermore, this is consistent with the broader literature, which highlights that structural and cultural barriers are more influential than individual characteristics in determining the success of Lean adoption.

These findings are also consistent with the literature, which identifies the main barriers to Lean implementation in healthcare as predominantly organisational and sociocultural. Specifically, resistance to change, limited staff engagement, and poor understanding of Lean principles and benefits represent recurring obstacles [50, 51]. Moreover, these issues are intertwined with structural characteristics typical of healthcare organisations. For example, “siloed” work and strong hierarchical models, in which high professional autonomy -especially among physicians- can hinder processes of standardisation and interdisciplinary collaboration [50, 52].

Deepening this perspective, the literature highlights how dynamics of “baronies,” professional tribalism, and scepticism towards an approach perceived as industrial in origin constitute additional significant barriers to the adoption and sustainability of Lean [50]. At a more serious level, these obstacles reflect difficulties in promoting shared and lasting cultural change, often associated with insufficient leadership engagement [53, 54]. Conversely, active oversight support, together with staff training and skills development, is recognised as one of the key enabling factors for effective Lean implementation [51, 54].

Overall, the evidence suggests that difficulties in adopting Lean are mainly attributable to systemic factors related to organisational culture, hierarchical structure, and leadership quality, rather than to individual characteristics. Consequently, the introduction of Lean tools, in the absence of parallel investments in training, staff engagement, and leadership development, is unlikely to result in sustainable change.

Our study reveals a paradox: very high caregiver satisfaction exists despite substantial operational inefficiencies. Communication and staff interactions outweigh waiting times and schedule adherence in shaping perceived quality in paediatric settings, underscoring the dominance of relational care in the patient experience. By focusing on a paediatric DH and examining staff perceptions alongside process performance, our results reinforce the primary role of organisational and cultural factors in Lean adoption.

### Strengths and limitations

The study demonstrates several notable strengths that contribute to its methodological robustness. Its design, which integrates process data, patient-reported outcomes, and staff perspectives, enables a comprehensive understanding of how the DH operates. The systematic application of VSM, supported by real-time time tracking, provides a precise depiction of workflow dynamics. High participation rates among both caregivers and staff further reinforce the reliability of the ouputs, while the involvement of an independent clinical epidemiology unit in conducting the statistical analyses adds an additional layer of credibility.

At the same time, certain limitations must be acknowledged. Firstly, because the research was conducted in a single tertiary paediatric centre, its generalisability to other settings remains limited. The study did not include clinical outcomes or measures of treatment effectiveness, which restricts the scope of its conclusions. Although representativeness was assessed using 2023 DH activity data, families facing literacy challenges or language barriers may still be underrepresented. Moreover, the cross-sectional nature of the design prevents any causal interpretations.

### Implications for policy, practice and research

The findings indicate that effective Lean implementation in paediatrics requires investment in both operational systems and relational competencies. This approach should enhance appointment reliability, reduce administrative barriers, and allow flexibility for complex cases. Frontline staff engagement in the design of improvements is essential.

Future research should expand the diagnostic framework across multiple centres, link operational changes to clinical outcomes, and explore the lasting impacts of Lean training on staff and workflow. Tailored strategies are needed for vulnerable families whose care needs differ from those assessed by standard protocols.

## Conclusions

This study evaluates organisational processes, caregiver experience, and staff perceptions in a paediatric DH using Lean principles.

The findings highlight a key tension: despite significant inefficiency, caregiver satisfaction remained high due to strong interpersonal communication and staff engagement. Efficiency metrics alone do not capture quality in paediatric care.

Healthcare professionals responded positively to Lean, especially with training, but identified barriers in organisational support and change management. Successful Lean adoption needs leadership, participation, and focus on professional culture.

The study shows diagnostic assessments help tailor Lean initiatives, guiding the balance between efficiency and family-centred care. Lean should adapt to paediatric DHs, not prescribe uniform standardisation.

At the same time, healthcare professionals generally viewed Lean positively, especially when they have been previously trained. However, they pointed out ongoing challenges like a lack of organisational support and change management. These insights demonstrate that successfully implementing Lean requires not only technical tools but also strong leadership, staff involvement, and consideration of professional culture.

Overall, the study reveals that diagnostic assessments can help guide the design of future Lean projects by separating avoidable waste from necessary clinical flexibility. Instead of insisting on uniform standardisation, Lean in paediatric DHs should be adjusted to balance efficiency, family-centred care, and staff engagement.

## Data Availability

The data underlying this article cannot be shared publicly due to the information governance requirements of the participating institution. Data will be made available from the corresponding author on reasonable request and with permission from the Institute for Maternal and Child Health—IRCCS *“Burlo Garofolo”*, Trieste, Italy.
